# Women’s awareness of breast cancer symptoms: a national cross-sectional study from Palestine

**DOI:** 10.1186/s12889-022-13224-7

**Published:** 2022-04-21

**Authors:** Mohamedraed Elshami, Ibrahim Al-Slaibi, Roba Jamal Ghithan, Mohammed Alser, Nouran Ramzi Shurrab, Islam Osama Ismail, Ibtisam Ismail Mahfouz, Aseel AbdulQader Fannon, Malak Ayman Qawasmi, Mona Radi Hawa, Narmeen Giacaman, Manar Ahmaro, Heba Mahmoud Okshiya, Rula Khader Zaatreh, Wafa Aqel AbuKhalil, Faten Darwish Usrof, Noor Khairi Melhim, Ruba Jamal Madbouh, Hala Jamal Abu Hziema, Raghad Abed-Allateef Lahlooh, Sara Nawaf Ubaiat, Nour Ali Jaffal, Reem Khaled Alawna, Salsabeel Naeem Abed, Bessan Nimer Abuzahra, Aya Jawad Abu Kwaik, Mays Hafez Dodin, Raghad Othman Taha, Dina Mohammed Alashqar, Roaa Abd-alfattah Mobarak, Tasneem Smerat, Nasser Abu-El-Noor, Bettina Bottcher

**Affiliations:** 1 Division of Surgical Oncology, Department of Surgery, University Hospitals Cleveland Medical Center, Cleveland, OH USA; 2 Ministry of Health, Gaza, Palestine; 3 Almakassed Hospital, Jerusalem, Palestine; 4 Faculty of Medicine, Al-Quds University, Jerusalem, Palestine; 5 Faculty of Medicine, Al Azhar University-Gaza, Gaza, Palestine; 6 Faculty of Medicine, Islamic University of Gaza, Gaza, Palestine; 7 Department of Medical Laboratory Sciences, Hebron University, Hebron, Palestine; 8 Tulkarem Governmental Hospital, Tulkarem, Palestine; 9 Al-Shifa Hospital, Gaza, Palestine; 10 Caritas Baby Hospital, Bethlehem, Palestine; 11 Department of Medical Laboratory Sciences, Faculty of Sciences, Islamic University of Gaza, Gaza, Palestine; 12 Department of Pharmacy, An-Najah National University, Nablus, Palestine; 13 Faculty of Dentistry, Al-Quds University, Jerusalem, Palestine; 14 Faculty of Medicine and Health Sciences, Palestine Polytechnic University, Hebron, Palestine; 15 Faculty of Nursing, Islamic University of Gaza, Gaza, Palestine

**Keywords:** Breast cancer, Early detection, Survival, Symptom, Awareness, Early presentation, Health education, Palestine

## Abstract

**Background:**

Early diagnosis is crucial to reduce the morbidity and mortality associated with breast cancer (BC). Awareness of BC symptoms plays a key role in this. This study aimed to evaluate the Palestinian women’s awareness of BC symptoms and determine factors associated with good awareness.

**Methods:**

This was a national cross-sectional study conducted from July 2019 to March 2020 in Palestine. Convenience sampling was used to recruit adult women from hospitals, primary healthcare centers, and public spaces located in 11 governorates. A translated-into-Arabic version of the validated BC awareness measure was utilized for data collection. The awareness level was categorized based on the number of symptoms recognized into: poor (0 to 4), fair (5 to 9), and good (10 to 13).

**Results:**

Of 6269 approached, 5434 participants completed the questionnaire (response rate = 86.7%). A total of 5257 questionnaires were included in the analysis: 2551 from the Gaza Strip and 2706 from the West Bank and Jerusalem (WBJ). Participants living in the WBJ were more likely to be older, have higher monthly income, and suffer from more chronic diseases than participants living in the Gaza Strip.

The most frequently identified BC symptom was ‘lump or thickening in the breast’ (*n* = 4887, 92.9%) followed by ‘lump or thickening under the armpit’ (*n* = 4394, 83.6%). The least frequently identified symptoms were ‘pulling in of the nipple’ (*n* = 2665, 50.7%) and ‘change in the position of the nipple’ (*n* = 2710, 51.6%).

A total of 2191 participants (41.7%) demonstrated good awareness of BC symptoms. Participants from the Gaza Strip were more likely than participants from the WBJ to have good awareness (47.0.0% vs. 36.7%). On the multivariable analysis, being ≥ 40 years, completing a post-secondary education, knowing someone with cancer, and visiting hospitals and primary healthcare centers were all associated with an increase in the likelihood of having good awareness. However, living in the WBJ was associated with a decrease in the likelihood of having good awareness.

**Conclusion:**

Less than half of women included in this study showed good awareness of BC symptoms. More targeted educational interventions are needed to promote Palestinian women’s awareness of BC symptoms to facilitate early diagnosis.

**Supplementary Information:**

The online version contains supplementary material available at 10.1186/s12889-022-13224-7.

## Introduction

Breast cancer (BC) is the most common cancer among women worldwide with 2.26 million new cases diagnosed in 2020 accounting for 24.5% of all cancers in women [1]. BC was responsible for more than 600,000 deaths in 2020; making it the leading cause of cancer-related deaths among women [2]. High-income countries have higher incidence rates of BC than low- and middle-income countries. However, low- and middle-income countries have higher mortality rates [3]. The age-standardized incidence and mortality rates of BC in Western Asia are 46.6 and 16.0 per 100,000 females, respectively. Palestine has higher incidence and mortality rates of 53.5 and 22.6 per 100,000 females, respectively [2, 4]. BC is considered a major public health concern in Palestine, where it is the most common cancer among females and has the second highest mortality rate (12.3%) after lung cancer (17.3%) [4].

BC is highly treatable if detected early through screening programs [5, 6]. Raising the awareness of women about the warning signs and symptoms of BC to encourage early seeking to medical advice could be another effective method for early detection [6]. This could be especially important in low-resource settings, such as Palestine [7–9]. In Palestine, women are first invited to undergo screening mammography at the age of 40, where they can access screening services free of discharge or at very low cost [10]. Nonetheless, some reports showed low awareness of the availability and the uptake of BC screening [7–9, 11].

A previous study demonstrated low awareness of BC in the Gaza Strip [11]. However, there is still an unmet need to investigate the national awareness of BC in Palestine. Creating a baseline awareness level may help future education interventions to be more efficient and to measure the magnitude of change in BC awareness. Another point to consider while designing these interventions is the nature of BC symptoms. BC symptoms can vary significantly but can be categorized into three main categories: breast symptoms, nipple symptoms, and other symptoms. Previous studies showed that breast symptoms were the most frequently presenting symptoms of BC followed by nipple symptoms and other symptoms [12]. Consequently, education interventions should be tailored to address the differing nature of BC symptoms and their role in early presentation. This could be facilitated by evaluating the awareness of each of these categories.

This study aimed to: i) assess the awareness level of BC symptoms among women in Palestine, ii) compare the awareness level between the two major areas in Palestine: the Gaza Strip as well as the West Bank and Jerusalem (WBJ), and iii) identify the factors associated with good awareness of BC symptoms.

## Materials and methods

### Study design and population

This study was a national cross-sectional study conducted from July 2019 to March 2020. The study population was Palestinian women aged 18 years and over. Recruitment of participants was done from among female visitors to Palestinian governmental hospitals, primary healthcare centers and public spaces, including malls, markets, gardens, restaurants, churches, mosques, and transportation stations. Recruitment took place in the two main geographical areas of Palestine, the Gaza Strip and the WBJ. Excluded from taking part in the study were women with a citizenship other than Palestinian, women working or studying in the field of health, healthcare and medicine, as well as those visiting oncology departments or clinics.

### Sampling methods

Convenience sampling was used to recruit eligible women from the designated data collection sites, governmental hospitals, primary healthcare centers, and public spaces located in 11 governorates across Palestine. This was intended to represent the diversity of the Palestinian community in the study cohort. In 2019, the estimated female population (≥ 15 years) was 947,100 females in the WBJ and 587,271 females in the Gaza Strip [13]. With a confidence level of 95.0% and a margin of error of 3.0%, a total minimum sample size of 2132 was needed (1066 for each of the WBJ and the Gaza Strip).

### Questionnaire and data collection

A modified version of the Breast Cancer Awareness Measure (BCAM) was used to collect data. The BCAM is a validated tool that was designed to measure the public awareness of BC [14]. The original BCAM was first translated into Arabic by two bilingual experts and then back-translated into English by another two different bilingual experts. The Arabic version of the BCAM was evaluated for clinical relevance and accuracy of translation by five experts in the field of BC, public health, and survey design. This was followed by running a pilot study (*n* = 35) to assess the clarity of questions in the Arabic version of the BCAM. The internal consistency of the Arabic BCAM was tested using Cronbach’s Alpha that reached an acceptable value of 0.753.

The questionnaire included two sections. The first section described the sociodemographic factors of study participants including age, menarche, parity, highest level of education, occupation, monthly income, marital status, place of residency, having a chronic disease, and knowing someone with cancer. The second section evaluated the participant’s awareness of 13 BC symptoms. Of the 13 BC symptoms, 11 were adopted from the original BCAM [14] and ‘extreme generalized fatigue’ as well as ‘unexplained weight loss’ have been added to the questionnaire since they were included in other forms of Cancer Awareness Measure [15–18], and it was thought that it would be helpful to include them in the context of BC. To minimize the possibility of participants answering questions randomly, the original questions evaluating the recognition of BC symptoms with yes/no/do not know responses were modified into a 5-point Likert scale (1 = strongly disagree, 5 = strongly agree). Meanwhile, the participants’ responses were subsequently converted to correct/incorrect responses similar to what was done in previous studies [19–24].

For data collection, an electronic tool ‘Kobo Toolbox’ was used [14]. This secure tool can be used offline and online through smart phones. Participants were invited to complete the questionnaire in a face-to-face interview. Female data collectors with a medical background received a special training on how to use Kobo Toolbox and how to approach potential participants in the waiting areas at hospitals, primary healthcare centers, and public spaces on a daily basis. The inclusion of female data collectors was intended to minimize the possibility of women feeling embarrassed to answer some sensitive questions. Securing privacy was part of the training received and was carefully considered, where recruited women were interviewed in private at the designated place. In addition, all interviews were completed with the presence of the interviewer only.

### Statistical analysis

The age of 40 is when women are first invited to undergo BC screening in Palestine [10]. Therefore, the continuous variable of age was categorized into two distinct groups using this cutoff: 18–39 years and ≥ 40 years. Menarche was categorized into three categories: early (≤ 11 years), normal (11–15 years), and late (≥ 16 years) [25]. Parity was also categorized into three categories: nulliparity, low multiparity [2–4], and grand multiparity (≥ 5) [26]. The minimum wage in the Palestinian community is 1450 NIS (about $450) [27]. As a result, this was selected as a cutoff to categorize the monthly income into two categories: < 1450 NIS and ≥ 1450 NIS.

Continuous non-normally distributed variables were described using the median and interquartile range (IQR). Categorical variables were described using frequencies and percentages. Baseline comparisons between the Gaza Strip vs. the WBJ were performed using Pearson's Chi-square test if the variable was categorical or Kruskal–Wallis test if it was continuous.

For questions based on the 5-point Likert scale, ‘strongly agree’ and ‘agree’ were considered to be correct answers whereas ‘strongly disagree’, ‘disagree’, and ‘not sure’ were considered to be incorrect answers. BC symptoms were further categorized into three categories: breast symptoms, nipple symptoms, and other symptoms. Recognition of each of the BC symptoms was described using frequencies and percentages with comparisons made by Pearson's Chi-Square test. This was followed by running bivariable and multivariable logistic regression analyses. The model of the multivariable analyses adjusted for age-group, educational level, occupation, monthly income, residency, having a chronic disease, knowing someone with cancer, marital status, and site of data collection. This model was determined based on other previous studies [11, 28–30]. Results of bivariable logistic regression analyses were provided in Additional file 1.

To evaluate the participants’ awareness level of BC symptoms, a previously used scoring system was also utilized in this study [19–24]. For each correctly recognized BC symptom, the participant was given one point. The total score (ranging from 0 to 13) was calculated and categorized into three categories based on the number of BC symptoms recognized: poor (0 to 4), fair (5 to 9), and good (10 to 13). Comparisons in the awareness level between the Gaza Strip vs. the WBJ were performed using Pearson's Chi-Square test. Bi- and multi-variable logistic regression analyses were also used to test the association between participant characteristics with having a good awareness level.

Sensitivity analyses were performed and included women who were married, divorced, or widowed. Bi- and multi-variable logistic regression analyses were utilized to examine the association between participant characteristics and displaying good awareness. The multivariable models adjusted for the same factors included in the main analyses in addition to parity. Results of the sensitivity analyses were provided in Additional file 1.

Complete case analysis was used to handle missing data, which were completely at random. Data were analyzed using Stata software version 16.0 (StataCorp, College Station, Texas, United States).

## Results

### Participant characteristics

Of the 6269 approached participants, 5434 participants completed the questionnaire (response rate = 86.7%). A total of 5257 questionnaires were included in the analysis (13 excluded and 164 had missing data): 2551 from the Gaza Strip and 2706 from the WBJ. The median age [IQR] for all participants was 31.0 years [24.0, 43.0] (Table 1). Participants living in the WBJ were more likely to be older, have higher monthly income, know someone with cancer, and suffer from more chronic diseases than participants living in the Gaza Strip.

**Table 1:** Characteristics of study participants

Characteristic	Total (*n* = 5257)	Gaza Strip (*n* = 2551)	WBJ (*n* = 2706)	*P*-value
**Age**, median [IQR]	31.0 [24.0, 43.0]	30.0 [24.0, 40.0]	33.0 [24.0, 45.0]	< 0.001
**Age group**, n (%)				
18 to 39	3615 (68.8)	1859 (72.9)	1756 (64.9)	< 0.001
40 or older	1642 (31.2)	692 (27.1)	950 (35.1)	
**Menarche**, n (%)				
Normal (11–15 years)	4608 (87.7)	2237 (87.7)	2371 (87.6)	0.003
Early (≤ 10 years)	72 (1.4)	21 (0.8)	51 (1.9)	
Late (≥ 16 years)	577 (10.9)	293 (11.5)	284 (10.5)	
Parity*, n (%)				
Nulliparity	306 (7.7)	142 (7.4)	164 (8.1)	0.69
Low multiparity	1898 (48.0)	923 (47.9)	975 (48.0)	
Grand multiparity	1752 (44.3)	860 (44.7)	892 (43.9)	
**Educational level**, n (%)				
Secondary or below	3030 (57.6)	1457 (57.1)	1573 (58.1)	0.46
Post-secondary	2227 (42.4)	1094 (42.9)	1133 (41.9)	
**Occupation**, n (%)				
Unemployed/home duties	3568 (67.9)	1868 (73.2)	1700 (62.8)	< 0.001
Employed	1052 (20.0)	380 (14.9)	672 (24.9)	
Retired	13 (0.3)	5 (0.2)	8 (0.3)	
Student	624 (11.8)	298 (11.7)	326 (12.0)	
**Monthly income ≥ 1450 NIS**, n (%)	3055 (58.1)	716 (28.1)	2339 (86.4)	< 0.001
**Having a chronic disease**, n (%)	1058 (20.1)	397 (15.6)	661 (24.4)	< 0.001
**Knowing someone with cancer**, n (%)	2520 (47.9)	1083 (42.5)	1437 (53.1)	< 0.001
Breast and/or ovarian cancer^#^	1026 (40.7)	491 (45.3)	535 (37.2)	
Other cancers^#^	1209 (48.0)	483 (44.6)	726 (50.5)	
Both^#^	285 (11.3)	109 (10.1)	176 (12.3)	
**Marital status**, n (%)				
Single	1301 (24.8)	626 (24.5)	675 (24.9)	< 0.001
Married	3658 (69.6)	1812 (71.0)	1846 (68.3)	
Divorced/Widowed	298 (5.6)	113 (4.5)	185 (6.8)	
**Site of data collection**				
Public spaces, n (%)	1821 (34.6)	809 (31.7)	1012 (37.4)	< 0.001
Hospitals, n (%)	2116 (40.3)	919 (36.0)	1197 (44.2)	
Primary healthcare centers, n (%)	1320 (25.1)	823 (32.3)	497 (18.4)	

*N* number of participants, *IQR* interquartile range, *WBJ* West Bank and Jerusalem

^*^The denominator is the number of married, divorced, or widowed women

^#^The denominator is the number of women who knew some with cancer at the time of the interview

### Good awareness and its associated factors

A total of 2191 participants (41.7%) demonstrated good awareness of BC symptoms (Table 2). Participants from the Gaza Strip were more likely than participants from the WBJ to have good awareness (47.0.0% vs. 36.7%).

**Table 2:** Awareness levels of breast cancer symptoms among study participants

Level	Total (*n* = 5257) n (%)	Gaza Strip (*n* = 2551) n (%)	WBJ (*n* = 2706) n (%)	*P*-value
Poor	559 (10.6)	256 (10.0)	303 (11.2)	< 0.001
Fair	2507 (47.7)	1098 (43.0)	1409 (52.1)	
Good	2191 (41.7)	1197 (47.0)	994 (36.7)	

*N* number of participants, *WBJ* West Bank and Jerusalem

On the multivariable analysis, being 40 years or older, completing a post-secondary education, knowing someone with cancer, and visiting hospitals and primary healthcare centers were all associated with an increase in the likelihood of having a good awareness level of BC symptoms (Table 3). However, living in the WBJ was associated with a decrease in the likelihood of having good awareness.

**Table 3:** The association between having a good awareness of breast cancer symptoms and participant characteristics

Characteristic	Good knowledge			
	**COR (95% CI)**	***P*-value**	AOR (95% CI)*	***P*-value**
**Age group**				
18 to 39	Ref	Ref	Ref	Ref
40 or older	1.36 (1.21–1.53)	<0.001	1.55 (1.35–1.80)	<0.001
**Educational level**				
Secondary or below	Ref	Ref	Ref	Ref
Post-secondary	1.21 (1.09–1.36)	0.001	1.44 (1.26–1.65)	<0.001
**Occupation**				
Unemployed/home duties	Ref	Ref	Ref	Ref
Employed	1.08 (0.94–1.24)	0.27	1.18 (1.00–1.39)	0.052
Retired	0.88 (0.29–2.69)	0.82	0.54 (0.17–1.68)	0.29
Student	0.92 (0.78–1.10)	0.36	1.24 (0.99–1.56)	0.06
**Monthly income**				
< 1450 NIS	Ref	Ref	Ref	Ref
≥ 1450 NIS	0.77 (0.69–0.86)	<0.001	0.88 (0.76–1.03)	0.10
**Marital status**				
Single	Ref	Ref	Ref	Ref
Married	1.16 (1.02–1.31)	0.029	1.02 (0.86–1.20)	0.84
Divorced/Widowed	1.23 (0.95–1.58)	0.11	1.04 (0.78–1.39)	0.78
**Residency**				
Gaza Strip	Ref	Ref	Ref	Ref
WBJ	0.66 (0.59–0.73)	<0.001	0.68 (0.59–0.79)	<0.001
**Having a chronic disease**				
No	Ref	Ref	Ref	Ref
Yes	1.11 (0.97–1.27)	0.14	1.00 (0.85–1.17)	0.99
**Knowing someone with cancer**				
No	Ref	Ref	Ref	Ref
Yes	1.12 (1.00–1.24)	0.052	1.21 (1.08–1.36)	0.001
**Site of data collection**				
Public Spaces	Ref	Ref	Ref	Ref
Hospitals	1.56 (1.37–1.77)	<0.001	1.70 (1.48–1.96)	0.001
Primary healthcare centers	1.86 (1.61–2.15)	<0.001	1.96 (1.68–2.29)	<0.001

*COR* crude odds ratio, *AOR* adjusted odds ratio, *CI* confidence interval, *WBJ* West Bank and Jerusalem

^*^Adjusted for age-group, educational level, occupation, monthly income, marital status, residency, having a chronic disease, knowing someone with cancer, and site of data collection

### Recognition of BC symptoms

Among all participants, breast symptoms were more often recognized than nipple symptoms. The most frequently identified BC symptom was ‘lump or thickening in the breast’ (*n* = 4887, 92.9%) followed by ‘lump or thickening under the armpit’ (*n* = 4394, 83.6%) (Table 4). These symptoms were also the most identified symptoms in both the Gaza Strip and the WBJ. The least frequently identified symptoms were ‘pulling in of the nipple’ (*n* = 2665, 50.7%) and ‘change in the position of the nipple’ (*n* = 2710, 51.6%). These symptoms were also the least identified symptoms in both the Gaza Strip and the WBJ.

**Table 4:** Recognition of breast cancer symptoms

Category of symptoms	Symptom	Total (*n* = 5257) n (%)	Gaza Strip *n* = 2551) n (%)	WBJ (*n* = 2706) n (%)	*P*-value
**Breast symptoms**	A lump or thickening in the breast	4887 (92.9)	2398 (94.0)	2489 (92.0)	0.004
	Pain in one of the breasts or armpits	3064 (58.3)	1479 (58.0)	1585 (58.6)	0.66
	Puckering or dimpling of the breast skin	2968 (56.5)	1517 (59.5)	1451 (53.6)	< 0.001
	Redness of the breast skin	2945 (56.0)	1559 (61.1)	1386 (51.2)	< 0.001
**Nipple symptoms**	Discharge or bleeding from the nipple	3785 (72.0)	1862 (73.0)	1923 (71.1)	0.12
	Nipple rash	3063 (58.3)	1604 (62.9)	1459 (53.9)	< 0.001
	Change in the position of the nipple	2710 (51.6)	1402 (55.0)	1308 (48.3)	< 0.001
	Pulling in of the nipple	2665 (50.7)	1400 (54.9)	1265 (46.7)	< 0.001
**Other symptoms**	Lump or thickening under the armpit	4394 (83.6)	2161 (84.7)	2233 (82.5)	0.032
	Changes in the shape of the breast or nipple	4059 (77.2)	2028 (79.5)	1985 (73.4)	< 0.001
	Changes in the size of the breast or nipple	4013 (76.3)	2065 (80.9)	1994 (73.7)	< 0.001
	Unexplained weight loss	3152 (60.0)	1544 (60.5)	1608 (59.4)	0.42
	Extreme generalized fatigue	3054 (58.1)	1557 (61.0)	1497 (55.3)	< 0.001

*N* number of participants, *WBJ* West Bank and Jerusalem

### Association between recognizing breast symptoms and participant characteristics

On the multivariable analysis, women who had benefitted from post-secondary education were more likely to identify all breast symptoms (Table 5). Additionally, women recruited from hospitals or primary healthcare centers were more likely than women recruited from public spaces to recognize three out of the four breast symptoms. In contrast, women residing in the WBJ were less likely than women residing in the Gaza Strip to recognize three out of the four breast symptoms.

**Table 5:** Multivariable logistic regression analyzing the association between recognizing breast symptoms and participant characteristics

characteristic	A lump or thickening in the breast		Pain in one of the breasts or armpits		Puckering or dimpling of the breast skin		Redness of the breast skin	
	AOR (95% CI)*	***P*-value**	AOR (95% CI)*	***P*-value**	AOR (95% CI)*	***P*-value**	AOR (95% CI)*	***P*-value**
**Age group**								
18 to 39	Ref	Ref	Ref	Ref	Ref	Ref	Ref	Ref
40 or older	1.18 (0.89–1.57)	0.24	0.80 (0.69–0.92)	0.002	1.52 (1.32–1.76)	<0.001	1.47 (1.27–1.69)	<0.001
**Educational level**								
Secondary or below	Ref	Ref	Ref	Ref	Ref	Ref	Ref	Ref
Post-secondary	1.94 (1.49–2.53)	<0.001	1.26 (1.10–1.44)	0.001	1.51 (1.32–1.73)	<0.001	1.23 (1.08–1.41)	0.002
**Occupation**								
Unemployed/home duties	Ref	Ref	Ref	Ref	Ref	Ref	Ref	Ref
Employed	0.85 (0.62–1.18)	0.34	0.85 (0.73–1.00)	0.054	1.08 (0.92–1.28)	0.33	0.98 (0.84–1.15)	0.83
Retired	0.38 (0.05–3.10)	0.37	4.40 (0.96–20.06)	0.06	1.54 (0.41–5.74)	0.52	0.65 (0.21–1.97)	0.45
Student	0.84 (0.56–1.26)	0.40	1.04 (0.83–1.31)	0.73	1.26 (1.01–1.58)	0.041	1.12 (0.90–1.40)	0.32
**Monthly income**								
< 1450 NIS	Ref	Ref	Ref	Ref	Ref	Ref	Ref	Ref
≥ 1450 NIS	1.87 (1.40–2.48)	<0.001	0.95 (0.82–1.10)	0.52	1.11 (0.96–1.29)	0.17	0.81 (0.70–0.94)	0.005
**Residency**								
Gaza Strip	Ref	Ref	Ref	Ref	Ref	Ref	Ref	Ref
WBJ	0.45 (0.34–0.61)	<0.001	1.15 (1.00–1.32)	0.06	0.73 (0.63–0.84)	<0.001	0.75 (0.65–0.86)	<0.001
**Having a chronic disease**								
No	Ref	Ref	Ref	Ref	Ref	Ref	Ref	Ref
Yes	1.02 (0.75–1.39)	0.89	0.91 (0.78–1.07)	0.26	0.94 (0.81–1.11)	0.47	0.97 (0.83–1.14)	0.73
**Knowing someone with cancer**								
No	Ref	Ref	Ref	Ref	Ref	Ref	Ref	Ref
Yes	1.46 (1.17–1.83)	0.001	0.94 (0.84–1.05)	0.25	1.21 (1.08–1.36)	0.001	1.11 (0.99–1.25)	0.07
**Marital status**								
Single	Ref	Ref	Ref	Ref	Ref	Ref	Ref	Ref
Married	1.40 (1.03–1.90)	0.029	0.87 (0.74–1.03)	0.11	0.86 (0.73–1.02)	0.08	1.15 (0.98–1.36)	0.09
Divorced/Widowed	2.27 (1.25–4.15)	0.007	0.93 (0.70–1.24)	0.63	0.87 (0.66–1.16)	0.35	0.85 (0.64–1.12)	0.25
**Site of data collection**								
Public spaces	Ref	Ref	Ref	Ref	Ref	Ref	Ref	Ref
Hospitals	1.40 (1.07–1.84)	0.016	1.06 (0.92–1.21)	0.43	1.84 (1.60–2.11)	<0.001	1.46 (1.28–1.68)	<0.001
Primary healthcare centers	0.90 (0.68–1.20)	0.48	1.36 (1.17–1.59)	<0.001	2.02 (1.73–2.36)	<0.001	1.50 (1.28–1.75)	<0.001

*AOR* adjusted odds ratio, *CI* confidence interval, *WBJ* West Bank and Jerusalem

^*^Adjusted for age-group, educational level, occupation, monthly income, marital status, residency, having a chronic disease, knowing someone with cancer, and site of data collection

Women aged ≥ 40 years were more likely than younger women (18–39 years) to recognize ‘puckering or dimpling of the breast skin’ (OR = 1.52, 95% CI: 1.32–1.76) and ‘redness of the breast skin’ (OR = 1.47, 95% CI: 1.27–1.69). However, women aged ≥ 40 years were less likely to recognize ‘pain in one of the breasts or armpits’ (OR = 0.80, 95% CI: 0.69–0.92).

### Association between recognizing nipple symptoms and participant characteristics

Women residing in the WBJ were less likely than women residing in the Gaza Strip to recognize all nipple symptoms (Table 6). On the contrary, women recruited from hospitals or primary healthcare centers were more likely than women recruited from public spaces to recognize all nipple symptoms. Additionally, women aged ≥ 40 years had a higher likelihood than younger women (18–39 years) to recognize all nipple symptoms.

**Table 6:** Multivariable logistic regression analyzing the association between recognizing nipple symptoms and participant characteristics

**Characteristic**	**Discharge or bleeding from the nipple**		**Nipple rash**		**Change in the position of the nipple**		**Pulling in of the nipple**	
	AOR (95% CI)*	***P*-value**	AOR (95% CI)*	***P*-value**	AOR (95% CI)*	***P*-value**	AOR (95% CI)*	***P*-value**
**Age group**								
18 to 39	Ref	Ref	Ref	Ref	Ref	Ref	Ref	Ref
40 or older	1.36 (1.16–1.59)	<0.001	1.25 (1.08–1.45)	0.002	1.46 (1.27–1.68)	<0.001	1.56 (1.35–1.80)	<0.001
**Educational level**								
Secondary or below	Ref	Ref	Ref	Ref	Ref	Ref	Ref	Ref
Post-secondary	1.36 (1.18–1.58)	<0.001	1.26 (1.10–1.43)	0.001	1.21 (1.06–1.38)	0.004	1.34 (1.17–1.53)	<0.001
**Occupation**								
Unemployed/home duties	Ref	Ref	Ref	Ref	Ref	Ref	Ref	Ref
Employed	1.33 (1.11–1.60)	0.002	1.00 (0.85–1.17)	0.99	1.03 (0.88–1.21)	0.72	1.27 (1.08–1.49)	0.004
Retired	1.30 (0.28–6.00)	0.73	1.79 (0.48–6.60)	0.38	3.30 (0.72–15.10)	0.12	2.04 (0.55–7.57)	0.28
Student	1.49 (1.17–1.91)	0.001	1.17 (0.94–1.47)	0.16	1.18 (0.95–1.47)	0.13	1.44 (1.15–1.80)	0.001
**Monthly income**								
< 1450 NIS	Ref	Ref	Ref	Ref	Ref	Ref	Ref	Ref
≥ 1450 NIS	1.47 (1.25–1.73)	<0.001	0.96 (0.82–1.11)	0.54	1.12 (0.97–1.30)	0.12	1.11 (0.96–1.29)	0.15
**Residency**								
Gaza Strip	Ref	Ref	Ref	Ref	Ref	Ref	Ref	Ref
WBJ	0.70 (0.59–0.82)	<0.001	0.70 (0.61–0.81)	<0.001	0.70 (0.60–0.80)	<0.001	0.66 (0.57–0.76)	<0.001
**Having a chronic disease**								
No	Ref	Ref	Ref	Ref	Ref	Ref	Ref	Ref
Yes	1.15 (0.97–1.38)	0.12	0.97 (0.83–1.14)	0.75	1.16 (1.00–1.36)	0.06	1.08 (0.92–1.26)	0.37
**Knowing someone with cancer**								
No	Ref	Ref	Ref	Ref	Ref	Ref	Ref	Ref
Yes	1.04 (0.92–1.17)	0.57	1.21 (1.08–1.36)	0.001	1.08 (0.97–1.21)	0.16	1.14 (1.02–1.28)	0.022
**Marital status**								
Single	Ref	Ref	Ref	Ref	Ref	Ref	Ref	Ref
Married	1.17 (0.98–1.40)	0.09	1.09 (0.92–1.28)	0.31	0.86 (0.73–1.01)	0.06	1.06 (0.90–1.25)	0.50
Divorced/Widowed	1.19 (0.87–1.63)	0.27	0.96 (0.73–1.28)	0.80	0.90 (0.68–1.19)	0.45	0.99 (0.75–1.32)	0.96
**Site of data collection**								
Public spaces	Ref	Ref	Ref	Ref	Ref	Ref	Ref	Ref
Hospitals	1.51 (1.30–1.75)	<0.001	1.37 (1.19–1.57)	<0.001	1.54 (1.35–1.77)	<0.001	1.50 (1.31–1.72)	<0.001
Primary healthcare centers	1.65 (1.39–1.95)	<0.001	1.40 (1.20–1.63)	<0.001	1.63 (1.40–1.90)	<0.001	1.86 (1.60–2.17)	<0.001

*AOR* adjusted odds ratio, *CI* confidence interval, *WBJ* West Bank and Jerusalem

^*^Adjusted for age-group, educational level, occupation, monthly income, marital status, residency, having a chronic disease, knowing someone with cancer, and site of data collection

**Table 7:** Multivariable logistic regression analyzing the association between recognizing other symptoms and participant characteristics

Characteristic	Lump or thickening under the armpit		Changes in the shape of the breast or nipple		Changes in the size of the breast or nipple		Unexplained weight loss		Extreme generalized fatigue	
	AOR (95% CI)*	***P*-value**	AOR (95% CI)*	***P*-value**	AOR (95% CI)*	***P*-value**	AOR (95% CI)*	***P*-value**	AOR (95% CI)*	***P*-value**
**Age group**										
18 to 39	Ref	Ref	Ref	Ref	Ref	Ref	Ref	Ref	Ref	Ref
40 or older	1.49 (1.21–1.83)	<0.001	1.35 (1.14–1.61)	0.001	1.30 (1.10–1.54)	0.002	1.25 (1.08–1.44)	0.003	1.00 (0.86–1.15)	0.96
**Educational level**										
Secondary or below	Ref	Ref	Ref	Ref	Ref	Ref	Ref	Ref	Ref	Ref
Post-secondary	1.66 (1.39–1.99)	<0.001	1.52 (1.30–1.78)	<0.001	1.35 (1.16–1.58)	<0.001	1.22 (1.06–1.39)	0.004	1.19 (1.05–1.36)	0.009
**Occupation**										
Unemployed/home duties	Ref	Ref	Ref	Ref	Ref	Ref	Ref	Ref	Ref	Ref
Employed	1.07 (0.85–1.34)	0.57	1.01 (0.83-1.23)	0.90	1.25 (1.03-1.51)	0.026	0.92 (0.78-1.08)	0.32	1.02 (0.87-1.20)	0.81
Retired	0.42 (0.09–1.94)	0.27	0.92 (0.20-4.26)	0.92	1.13 (0.25-5.20)	0.87	0.34 (0.11-1.05)	0.06	0.74 (0.25-2.24)	0.60
Student	0.95 (0.72–1.25)	0.72	1.09 (0.84–1.41)	0.53	1.09 (0.85–1.40)	0.50	1.02 (0.81–1.27)	0.89	1.18 (0.94–1.47)	0.15
**Monthly income**										
< 1450 NIS	Ref	Ref	Ref	Ref	Ref	Ref	Ref	Ref	Ref	Ref
≥ 1450 NIS	1.44 (1.18–1.76)	<0.001	1.27 (1.06–1.51)	0.009	1.14 (0.96–1.36)	0.13	0.97 (0.83–1.12)	0.66	0.86 (0.75–1.00)	0.051
**Residency**										
Gaza Strip	Ref	Ref	Ref	Ref	Ref	Ref	Ref	Ref	Ref	Ref
WBJ	0.62 (0.51–0.76)	<0.001	0.55 (0.46–0.65)	<0.001	0.62 (0.52–0.73)	<0.001	0.90 (0.78–1.04)	0.15	0.83 (0.72–0.96)	0.012
**Having a chronic disease**										
No	Ref	Ref	Ref	Ref	Ref	Ref	Ref	Ref	Ref	Ref
Yes	1.14 (0.91–1.43)	0.24	1.05 (0.87–1.27)	0.62	1.08 (0.90–1.30)	0.41	1.09 (0.92–1.28)	0.32	1.16 (0.99–1.36)	0.06
**Knowing someone with cancer**										
No	Ref	Ref	Ref	Ref	Ref	Ref	Ref	Ref	Ref	Ref
Yes	1.47 (1.26–1.71)	<0.001	1.21 (1.06–1.39)	0.005	1.08 (0.94–1.23)	0.27	1.33 (1.19–1.49)	<0.001	1.07 (0.95–1.20)	0.25
**Marital status**										
Single	Ref	Ref	Ref	Ref	Ref	Ref	Ref	Ref	Ref	Ref
Married	1.57 (1.27–1.93)	<0.001	1.15 (0.95-1.39)	0.16	1.19 (0.99-1.44)	0.07	1.06 (0.90-1.25)	0.47	0.98 (0.83-1.15)	0.77
Divorced/Widowed	1.56 (1.06–2.29)	0.025	0.84 (0.61–1.16)	0.29	1.02 (0.74–1.41)	0.91	1.35 (1.01–1.82)	0.043	0.92 (0.69–1.22)	0.56
**Site of data collection**										
Public spaces	Ref	Ref	Ref	Ref	Ref	Ref	Ref	Ref	Ref	Ref
Hospitals	1.52 (1.26–1.83)	<0.001	1.63 (1.39-1.92)	<0.001	1.51 (1.29-1.77)	0.001	1.62 (1.41-1.86)	<0.001	1.15 (1.00-1.32)	0.043
Primary healthcare centers	1.27 (1.04–1.56)	0.020	1.39 (1.16–1.66)	<0.001	1.28 (1.07–1.52)	0.007	1.08 (0.93–1.26)	0.34	1.00 (0.86–1.17)	0.99

*AOR* adjusted odds ratio, *CI* confidence interval, *WBJ *West Bank and Jerusalem

^*^Adjusted for age-group, educational level, occupation, monthly income, marital status, residency, having a chronic disease, knowing someone with cancer, and site of data collection

### Association between recognizing other BC symptoms and participant characteristics

Women residing in the WBJ were less likely than women residing in the Gaza Strip to recognize all other BC symptoms except ‘unexplained weight loss’ for which no difference was noticed (Table 7). In contrast, women recruited from hospitals and those with post-secondary education were more likely to recognize all other BC symptoms.

## Discussion

Good BC awareness is associated with early diagnosis, which leads to higher survival rates [5, 31–33]. Improving the awareness of BC symptoms could be especially critical in Palestine due to low resources and uptake of BC screening [7–9]. This study assessed the existing awareness of BC symptoms in Palestine to help in building strategies and establishing programs that work on raising the awareness of BC symptoms. Although several activities for this purpose were carried out [34, 35], the results of this study suggest that educational interventions should be tailored more to the context of the Palestinian community. This is in concordance with the findings of Sabi and colleagues in their study, where participants who attended cancer awareness campaigns did not show higher overall knowledge about cancer warning signs and risk factors [36]. The consensus at the 12^th^ Breast, Gynecological and Immuno-oncology International Cancer Conference in Egypt concluded that BC awareness campaigns should consider specific disease criteria and socioeconomic status of the country [37]. This is especially important for women in the Gaza Strip, where most women diagnosed with BC have advanced stages. Moreover, patients do not have access to adequate treatment options locally and movement restrictions impede their ability to travel outside to receive cancer care [38].

### Awareness level of BC symptoms

The low to moderate awareness of BC symptoms in this study is similar to the findings of Al-Mousa and colleagues among Jordanian women with 44.0% displaying good knowledge of BC symptoms [39]. Hassan and colleagues showed lower knowledge of BC in Egypt with 33.2% demonstrating good knowledge [40]. Other previous studies from non-Arab countries including China, Turkey, Nigeria, and Singapore also showed inadequate awareness of BC symptoms [29, 41–43]. A study conducted in Lebanon found a higher awareness of BC symptoms than of BC treatment options and curability. The authors explained that could be due to the focus of public campaigns on the detection of BC through its symptoms not the potential to cure BC [44]. Negative beliefs and worry about a potential BC diagnosis were also found to be barriers to early diagnosis in other studies [11, 20, 45] Future educational interventions should focus more on clarifying the link between the recognition of BC symptoms and early diagnosis leading to higher chances of curability [20]. In concordance with this study, previous studies found that women recognized breast symptoms, especially those with lump, more often than nipple symptoms [12, 46–48]. In fact, patients with non-lump symptoms were more likely to delay their medical visit [12]. This necessitates that the nature of BC symptoms should be included in the design of educational interventions aiming to raise the awareness of BC symptoms.

In comparison with another study looking at the awareness of cervical and ovarian cancer warning signs and symptoms among Palestinian women, good BC symptoms awareness in this study is relatively higher (41.7% vs. 27.4% and 17.4%, respectively) [21, 23]. This could be due to local awareness campaigns focusing on BC rather than other cancers. Quintanilha and colleagues demonstrated the efficiency of the ‘Pink October’ in increasing the interest of the Brazilian population in searching the internet about BC awareness [49]. Another contributing factor for the better awareness of BC symptoms could be the greater incidence of BC than other cancers, which may lead to higher chances that women could know someone with BC [1]. This may drive women to read more about BC.

### Factors associated with good awareness of BC symptoms

In line with this study, previous studies showed that higher education was associated with higher awareness of BC symptoms [29, 43, 48, 50–53]. This suggests that targeting women with curricula discussing health-related topics such as BC could be an effective strategy to raise their awareness [11]. In addition, similar to this study, a study conducted in Kuwait found that participants who knew someone diagnosed with cancer were more likely to recognize BC symptoms [54]. Knowing someone with cancer might make women feel more worried about having it themselves. This feeling may encourage women to search and ask about the BC symptoms, which will be reflected as a higher level of awareness about BC symptoms [21]. Further research is needed to explore the emotional and practical driving forces of women to learn more about BC symptoms and what sources they use to enrich their knowledge.

In this study, older women were more likely to have good awareness of BC symptoms. Older women have usually been more frequently exposed to healthcare professionals than younger women for maternity care as well as sexual and reproductive health purposes than younger women.

Such visits may help older women to accumulate more information about health-related topics including BC symptoms [21–24]. To further investigate this, a sensitivity analysis was performed among married, divorced, or widowed women and found no association between parity and displaying good awareness of BC symptoms (supplementary table 1). However, the finding that women recruited from hospitals or primary healthcare centers were more likely to have good awareness of BC symptoms (both in the main and sensitivity analyses) indicates that the exposure to healthcare professionals and other sources of information in healthcare facilities seems to play a role in shaping women’s health literacy. Governmental hospitals and primary healthcare centers can be attended free of charge by anyone with public health insurance or at very low cost by those who do not choose to pay for the publicly available low-cost health insurance. Therefore, a broad section of the Palestinian population can be met at governmental hospitals and primary healthcare centers. Women recruited from hospitals or primary healthcare centers display health-seeking behavior by their attendance of healthcare facilities. This might reflect an interest in their health and a greater general awareness of issues around health when compared to the group of women recruited from public spaces [11]. Therefore, both, more chances to have benefitted from education by healthcare professionals as well as their own health-seeking behavior, might have provided older women and those women visiting healthcare facilities with more opportunities to enhance their awareness of BC symptoms. Thus, exposure to health education and information appears to increase BC awareness and might be an important factor to improve early detection of BC and, hence, outcomes for women suffering from BC [32–34].

### Awareness of BC symptoms in the WBJ vs. the Gaza Strip

Women from the Gaza Strip were more likely than women from the WBJ to recognize BC symptoms. This could be explained by the lower exposure of women residing in the WBJ to healthcare professionals, therefore, they had lower chances to promote their health literacy [21]. The WBJ has checkpoints and restrictions on the internal movement, even between cities, leading to longer hours of delay to reach healthcare facilities accompanied with the fear and anxiety to come across checkpoints and settlements [55]. In addition, the WBJ has a wider geographical variation than the Gaza Strip and more women are living in rural areas in the WBJ than the Gaza Strip limiting their access to healthcare centers [56].

### Future directions

The results of this study highlight the substantial need to create sustainable educational interventions to raise Palestinian women’s awareness about BC symptoms. These interventions should adopt different strategies that aim to maximize the outreach especially to women living in underserved areas. This could be facilitated by running health education activities in coordination with the mobile healthcare clinics of the Palestinian Ministry of Health that are distributed across the WBJ [57]. Education programs should include the less known BC symptoms beyond the breast lump as well as the more positive outlook on prognosis with an early diagnosis in order to improve impact.

Involving healthcare professionals in these activities should also be considered [37]. This will necessitate training Palestinian healthcare professionals on how to deliver information about health topics, including BC, in a way that is appropriate to the health literacy of the public. In addition, a special training could be implemented to improve the communication skills of Palestinian healthcare professionals so that women would have more confidence to talk about their concerns if they recognized any possible BC symptoms. The diagnosis of BC could be stressful for women and training healthcare professionals on how to handle such situations could help reduce women’s stress especially that BC patients may rely on oncologists for their diagnosis-related emotional and social issues [58].

### Strengths and limitations

The major strengths of this study include the use of a translated version of the validated tool (BCAM) to assess women’s awareness of BC symptoms and the high response rate. In addition, the large sample size covering most geographical areas of Palestine may have generated the diversity of the Palestinian community in the study cohort. This study also has some limitations. The use of the convenience sampling may potentially limit the generalizability of the findings. However, the large number of study participants, the high response rate, and the diversity of geographical areas covered may mitigate this limitation. For example, the lower monthly income among women from the Gaza Strip than that of women from the WBJ mirrored the difference in the unemployment rates that are higher in the Gaza Strip than in the WBJ (47% vs. 16%) [27]. Moreover, the sociodemographics of the women included in this study were close to the demographics reported by other studies conducted in Palestine to assess awareness of various cancers [21–24]. Another limitation could be that the study included participants who did not experience actual BC symptoms and looked at their perceived knowledge. Finally, the recognition of BC symptoms was assessed while it might have been helpful to assess the recall of these symptoms as well.

## Conclusions

Less than half of study participants (41.7%) demonstrated good awareness of BC symptoms. Participants from the Gaza Strip were more likely to have good awareness than participants from the WBJ. The factors associated with good awareness included being 40 years or older, completing a post-secondary education, knowing someone with cancer, and visiting hospitals and primary healthcare centers. The most frequently identified BC symptom was ‘lump or thickening in the breast’ followed by ‘lump or thickening under the armpit’. Future educational interventions aiming to raise BC awareness should be tailored to the needs of women in Palestine.

## Supplementary Information

**Additional file 1.** 

**Additional file 2.** 

## Data Availability

The dataset used and analyzed during the current study is available from the corresponding author on reasonable request.

## Abbreviations

BC: Breast cancer
WBJ: West Bank and Jerusalem
BCAM: Breast cancer awareness measure
CI: Confidence interval
OR: Odds ratio
