# Supplementary material for: Women’s awareness of breast cancer symptoms: a national cross-sectional study from Palestine
Source: BMC Public Health. 2022 Apr 21;22:801. doi: 10.1186/s12889-022-13224-7 (PMC9027340; doi:10.1186/s12889-022-13224-7)
Supplement: Supplementary file 1 — Additional file 1. [file 12889_2022_13224_MOESM1_ESM.docx]

**Women’s Awareness of Breast Cancer Symptoms: A National Cross-sectional Study from Palestine**

Mohamedraed Elshami, MD^1,2^*, Ibrahim Al-Slaibi, MD^3^*, Roba Jamal Ghithan^4^*, Mohammed Alser, MD^2^*, Nouran Ramzi Shurrab^4^, Islam Osama Ismail^5^, Ibtisam Ismail Mahfouz^6^, Aseel AbdulQader Fannon^6^, Malak Ayman Qawasmi^7^, Mona Radi Hawa^8^, Narmeen Giacaman^4^, Manar Ahmaro^4^, Heba Mahmoud Okshiya^9^, Rula Khader Zaatreh^10^, Wafa Aqel AbuKhalil^4^, Faten Darwish Usrof^11^, Noor Khairi Melhim^12^, Ruba Jamal Madbouh^4^, Hala Jamal Abu Hziema^6^, Raghad Abed-Allateef Lahlooh^4^, Sara Nawaf Ubaiat^4^, Nour Ali Jaffal^4^, Reem Khaled Alawna^4^, Salsabeel Naeem Abed^6^, Bessan Nimer Abuzahra^4^, Aya Jawad Abu Kwaik^13^, Mays Hafez Dodin^4^, Raghad Othman Taha^4^, Dina Mohammed Alashqar^6^, Roaa Abd-alfattah Mobarak^4^, Tasneem Smerat, MSc^14^, Nasser Abu-El-Noor, PhD^15#^, Bettina Bottcher, MD, PhD^6#^

*Contributed equally as a first co-author.

^#^Contributed equally as a senior co-author.

^1^Division of Surgical Oncology, Department of Surgery, University Hospitals Cleveland Medical Center, Cleveland, OH, USA

^2^Ministry of Health, Gaza, Palestine.

^3^Almakassed Hospital, Jerusalem, Palestine.

^4^Faculty of Medicine, Al-Quds University, Palestine

^5^Faculty of Medicine, Al Azhar University-Gaza, Gaza, Palestine

^6^Faculty of Medicine, Islamic University of Gaza, Gaza, Palestine.

^7^Department of Medical Laboratory Sciences, Hebron University, Hebron, Palestine

^8^Tulkarem Governmental Hospital, Tulkarem, Palestine

^9^Al-Shifa Hospital, Gaza, Palestine

^10^Caritas Baby Hospital, Bethlehem, Palestine

^11^Faculty of Health Sciences Master of Medical Laboratory Sciences, Islamic University of Gaza, Gaza, Palestine

^12^Department of Pharmacy, An-Najah National University, Nablus, Palestine.

^13^Faculty of Dentistry, Al-Quds University, Jerusalem, Palestine

^14^Faculty of Medicine and Health Sciences, Palestine Polytechnic University, Hebron, Palestine

^15^Faculty of Nursing, Islamic University of Gaza, Gaza, Palestine.

**Corresponding author**

Mohamedraed Elshami, MD, MMSc

Division of Surgical Oncology

Department of Surgery

University Hospitals Cleveland Medical Center

11100 Euclid Avenue, Lakeside 7100

Cleveland, OH 44106
Phone: 832-245-6055

Email: [mohamedraed.elshami@gmail.com](mailto:mohamedraed.elshami@gmail.com)

Supplementary table 1: The association between participant characteristics and having a good awareness of breast cancer symptoms among married, divorced, or widowed women.

| **Characteristic** | **Good knowledge** | | | |
| --- | --- | --- | --- | --- |
|  | **COR (95% CI)** | **p-value** | **AOR (95% CI)*** | **p-value** |
| **Age group**  18 to 39  40 or older | Ref  1.33 (1.17-1.51) | Ref  0.001> | Ref  1.50 (1.27-1.78) | Ref  0.001> |
| **Parity**  Nulliparity  Low multiparity  Grand multiparity | Ref  0.95 (0.74-1.21)  1.10 (0.86-1.41) | Ref  0.66  0.44 | Ref  0.91 (0.71-1.18)  0.99 (0.75-1.29) | Ref  0.49  0.91 |
| **Educational level**  Secondary or below  Post-secondary | Ref  1.32 (1.15-1.50) | Ref  0.001> | Ref  1.53 (1.30-1.80) | Ref  0.001> |
| **Occupation**  Unemployed/home duties  Employed  Retired  Student | Ref  1.17 (0.99-1.38)  0.69 (0.21-2.30)  0.86 (0.52-1.40) | Ref  0.07  0.55  0.53 | Ref  1.14 (0.94-1.38)  0.42 (0.12-1.42)  0.98 (0.59-1.63) | Ref  0.18  0.16  0.94 |
| **Monthly income**  < 1450 NIS  ≥ 1450 NIS | Ref  0.78 (0.69-0.89) | Ref  0.001> | Ref  0.84 (0.70-1.00) | Ref  0.048 |
| **Marital status**  Married  Divorced/Widowed | Ref  1.06 (0.84-1.35) | Ref  0.61 | Ref  1.02 (0.79-1.31) | Ref  0.88 |
| **Residency**  Gaza Strip  WBJ | Ref  0.69 (0.61-0.79) | Ref  0.001> | Ref  0.76 (0.64-0.90) | Ref  0.001 |
| **Having a chronic disease**  No  Yes | Ref  1.06 (0.91-1.23) | Ref  0.44 | Ref  0.96 (0.81-1.14) | Ref  0.65 |
| **Knowing someone with cancer**  No  Yes | Ref  1.12 (0.99-1.27) | Ref  0.07 | Ref  1.21 (1.06-1.38) | Ref  0.004 |
| **Site of data collection**  Public Spaces  Hospitals  Primary healthcare centers | Ref  1.49 (1.27-1.75)  1.74 (1.46-2.07) | Ref  0.001>  0.001> | Ref  1.62 (1.38-1.91)  1.86 (1.55-2.23) | Ref  0.001>  0.001> |

COR= crude odds ratio, AOR= adjusted odds ratio, CI= confidence interval, WBJ= West Bank and Jerusalem.
* Adjusted for age-group, parity, educational level, occupation, monthly income, marital status, residency, having a chronic disease, knowing someone with cancer, and site of data collection.

| **characteristic**  Supplementary table 2: Multivariable logistic regression analyzing the association between participant characteristics and the recognition of breast symptoms among married, divorced, or widowed women. | **A lump or thickening in the breast** | | **Pain in one of the breasts or armpits** | | **Puckering or dimpling of the breast skin** | | **Redness of the breast skin** | |
| --- | --- | --- | --- | --- | --- | --- | --- | --- |
|  | **AOR (95% CI)*** | **p-value** | **AOR (95% CI)*** | **p-value** | **AOR (95% CI)*** | **p-value** | **AOR (95% CI)*** | **p-value** |
| **Age group**  18 to 39  40 or older | Ref  1.11 (0.79-1.55) | Ref  0.56 | Ref  0.84 (0.71-0.99) | Ref  0.038 | Ref  1.46 (1.23-1.73) | Ref  0.001> | Ref  1.53 (1.29-1.81) | Ref  0.001> |
| **Parity**  Nulliparity  Low multiparity  Grand multiparity | Ref  1.10 (0.68-1.78)  1.30 (0.77-2.18) | Ref  0.71  0.32 | Ref  0.87 (0.67-1.12)  0.79 (0.61-1.04) | Ref  0.27  0.09 | Ref  1.03 (0.80-1.33)  1.09 (0.83-1.42) | Ref  0.79  0.53 | Ref  0.90 (0.70-1.16)  0.87 (0.66-1.14) | Ref  0.43  0.30 |
| **Educational level**  Secondary or below  Post-secondary | Ref  2.23 (1.57-3.17) | Ref  0.001> | Ref  1.27 (1.08-1.49) | Ref  0.003 | Ref  1.57 (1.34-1.85) | Ref  0.001> | Ref  1.32 (1.12-1.55) | Ref  0.001 |
| **Occupation**  Unemployed/home duties  Employed  Retired  Student | Ref  0.81 (0.54-1.20)  0.33 (0.04-2.79)  1.04 (0.36-2.96) | Ref  0.29  0.31  0.94 | Ref  0.79 (0.65-0.96)  4.08 (0.88-18.89)  1.61 (0.93-2.81) | Ref  0.016  0.07  0.09 | Ref  1.12 (0.92-1.36)  1.41 (0.37-5.34)  1.09 (0.66-1.80) | Ref  0.25  0.62  0.73 | Ref  0.95 (0.78-1.15)  0.53 (0.17-1.66)  1.11 (0.67-1.83) | Ref  0.57  0.27  0.69 |
| **Monthly income**  < 1450 NIS  ≥ 1450 NIS | Ref  1.51 (1.06-2.15) | Ref  0.023 | Ref  1.02 (0.86-1.21) | Ref  0.84 | Ref  1.04 (0.87-1.24) | Ref  0.68 | Ref  0.81 (0.68-0.97) | Ref  0.021 |
| **Residency**  Gaza Strip  WBJ | Ref  0.48 (0.33-0.68) | Ref  0.001> | Ref  1.06 (0.90-1.26) | Ref  0.48 | Ref  0.76 (0.64-0.90) | Ref  0.001 | Ref  0.80 (0.67-0.95) | Ref  0.010 |
| **Having a chronic disease**  No  Yes | Ref  1.02 (0.73-1.43) | Ref  0.91 | Ref  0.90 (0.76-1.06) | Ref  0.22 | Ref  0.97 (0.82-1.15) | Ref  0.73 | Ref  0.96 (0.81-1.14) | Ref  0.62 |
| **Knowing someone with cancer**  No  Yes | Ref  1.38 (1.06-1.80) | Ref  0.016 | Ref  0.91 (0.80-1.04) | Ref  0.17 | Ref  1.17 (1.03-1.34) | Ref  0.016 | Ref  1.15 (1.01-1.31) | Ref  0.042 |
| **Marital status**  Married  Divorced/Widowed | Ref  1.63 (0.93-2.87) | Ref  0.09 | Ref  1.08 (0.84-1.39) | Ref  0.54 | Ref  1.00 (0.78-1.29) | Ref  0.98 | Ref  0.72 (0.56-0.93) | Ref  0.012 |
| **Site of data collection**  Public spaces  Hospitals  Primary healthcare centers  AOR= adjusted odds ratio, CI= confidence interval, WBJ= West Bank and Jerusalem. * Adjusted for age-group, parity, educational level, occupation, monthly income, marital status, residency, having a chronic disease, knowing someone with cancer, and site of data collection. | Ref  1.39 (1.00-1.93)  0.88 (0.62-1.24) | Ref  0.047  0.46 | Ref  1.12 (0.95-1.31)  1.46 (1.22-1.75) | Ref  0.17  0.001> | Ref  1.75 (1.49-2.05)  1.93 (1.61-2.31) | Ref  0.001>  0.001> | Ref  1.47 (1.25-1.73)  1.48 (1.24-1.77) | Ref  0.001>  0.001> |

| **Characteristic**  Table 3: Association between recognizing breast symptoms and participant characteristics.  Table 3: Association between recognizing breast symptoms and sociodemographic factors. | **Discharge or bleeding from the nipple** | | **Nipple rash** | | **Change in the position of the nipple** | | **Pulling in of the nipple** | |
| --- | --- | --- | --- | --- | --- | --- | --- | --- |
|  | **AOR (95% CI)*** | **p-value** | **AOR (95% CI)*** | **p-value** | **AOR (95% CI)*** | **p-value** | **AOR (95% CI)*** | **p-value** |
| **Age group**  18 to 39  40 or older | Ref  1.22 (1.01-1.47) | Ref  0.042 | Ref  1.17 (0.99-1.39) | Ref  0.06 | Ref  1.51 (1.28-1.78) | Ref  0.001> | Ref  1.47 (1.24-1.74) | Ref  0.001> |
| **Parity**  Nulliparity  Low multiparity  Grand multiparity | Ref  1.11 (0.85-1.46)  1.30 (0.97-1.73) | Ref  0.44  0.08 | Ref  0.82 (0.64-1.06)  0.88 (0.67-1.15) | Ref  0.13  0.35 | Ref  1.08 (0.84-1.38)  0.97 (0.75-1.27) | Ref  0.55  0.83 | Ref  0.87 (0.67-1.11)  0.97 (0.75-1.27) | Ref  0.26  0.85 |
| **Educational level**  Secondary or below  Post-secondary | Ref  1.47 (1.23-1.76) | Ref  0.001> | Ref  1.35 (1.15-1.59) | Ref  0.001> | Ref  1.23 (1.05-1.44) | Ref  0.009 | Ref  1.47 (1.25-1.72) | Ref  0.001> |
| **Occupation**  Unemployed/home duties  Employed  Retired  Student | Ref  1.46 (1.16-1.83)  1.15 (0.25-5.39)  1.10 (0.64-1.89) | Ref  0.001  0.86  0.73 | Ref  0.98 (0.81-1.19)  1.48 (0.39-5.56)  0.85 (0.52-1.40) | Ref  0.87  0.57  0.53 | Ref  1.04 (0.86-1.26)  3.00 (0.65-13.88)  0.83 (0.50-1.36) | Ref  0.66  0.16  0.46 | Ref  1.37 (1.13-1.66)  1.84 (0.49-6.94)  1.28 (0.78-2.10) | Ref  0.001  0.37  0.33 |
| **Monthly income**  < 1450 NIS  ≥ 1450 NIS | Ref  1.25 (1.03-1.52) | Ref  0.025 | Ref  1.02 (0.86-1.22) | Ref  0.80 | Ref  1.08 (0.91-1.29) | Ref  0.38 | Ref  0.98 (0.82-1.16) | Ref  0.79 |
| **Residency**  Gaza Strip  WBJ | Ref  0.80 (0.66-0.97) | Ref  0.026 | Ref  0.72 (0.60-0.85) | Ref  0.001> | Ref  0.75 (0.64-0.89) | Ref  0.001 | Ref  0.76 (0.64-0.90) | Ref  0.002 |
| **Having a chronic disease**  No  Yes | Ref  1.15 (0.95-1.40) | Ref  0.14 | Ref  1.03 (0.87-1.21) | Ref  0.77 | Ref  1.18 (1.00-1.40) | Ref  0.047 | Ref  1.04 (0.88-1.23) | Ref  0.66 |
| **Knowing someone with cancer**  No  Yes | Ref  1.06 (0.92-1.23) | Ref  0.41 | Ref  1.24 (1.08-1.41) | Ref  0.001 | Ref  1.05 (0.92-1.19) | Ref  0.47 | Ref  1.12 (0.98-1.27) | Ref  0.10 |
| **Marital status**  Married  Divorced/Widowed | Ref  1.01 (0.76-1.34) | Ref  0.94 | Ref  0.88 (0.69-1.14) | Ref  0.34 | Ref  1.01 (0.79-1.30) | Ref  0.91 | Ref  0.91 (0.70-1.16) | Ref  0.44 |
| **Site of data collection**  Public spaces  Hospitals  Primary healthcare centers  AOR= adjusted odds ratio, CI= confidence interval, WBJ= West Bank and Jerusalem. * Adjusted for age-group, parity, educational level, occupation, monthly income, marital status, residency, having a chronic disease, knowing someone with cancer, and site of data collection. | Ref  1.41 (1.18-1.68)  1.55 (1.27-1.88) | Ref  0.001>  0.001> | Ref  1.35 (1.15-1.58)  1.33 (1.11-1.59) | Ref  0.001>  0.002 | Ref  1.37 (1.17-1.61)  1.45 (1.21-1.73) | Ref  0.001>  0.001> | Ref  1.36 (1.16-1.59)  1.67 (1.39-1.99) | Ref  0.001>  0.001> |

Supplementary table 3: Multivariable logistic regression analyzing the association between participant characteristics and the recognition of nipple symptoms among married, divorced, or widowed women.

| **Characteristic**  Supplementary table 4: Multivariable logistic regression analyzing the association between participant characteristics and the recognition of other symptoms among married, divorced, or widowed women. | **Lump or thickening under the armpit** | | **Changes in the shape of the breast or nipple** | | **Changes in the size of the breast or nipple** | | **Unexplained weight loss** | | **Extreme generalized fatigue** | |
| --- | --- | --- | --- | --- | --- | --- | --- | --- | --- | --- |
|  | **AOR (95% CI)*** | **p-value** | **AOR (95% CI)*** | **p-value** | **AOR (95% CI)*** | **p-value** | **AOR (95% CI)*** | **p-value** | **AOR (95% CI)*** | **p-value** |
| **Age group**  18 to 39  40 or older | Ref  1.48 (1.17-1.89) | Ref  0.001 | Ref  1.31 (1.07-1.60) | Ref  0.010 | Ref  1.23 (1.01-1.50) | Ref  0.039 | Ref  1.20 (1.01-1.43) | Ref  0.034 | Ref  1.01 (0.86-1.20) | Ref  0.88 |
| **Parity**  Nulliparity  Low multiparity  Grand multiparity | Ref  1.12 (0.80-1.58)  1.02 (0.71-1.47) | Ref  0.51  0.91 | Ref  0.88 (0.65-1.19)  1.00 (0.72-1.38) | Ref  0.40  0.99 | Ref  0.88 (0.66-1.19)  0.96 (0.70-1.32) | Ref  0.42  0.80 | Ref  1.10 (0.86-1.42)  1.17 (0.89-1.53) | Ref  0.45  0.26 | Ref  1.02 (0.80-1.31)  1.00 (0.76-1.30) | Ref  0.87  0.98 |
| **Educational level**  Secondary or below  Post-secondary | Ref  1.59 (1.26-2.01) | Ref  0.001> | Ref  1.66 (1.36-2.02) | Ref  0.001> | Ref  1.36 (1.12-1.64) | Ref  0.002 | Ref  1.26 (1.07-1.48) | Ref  0.006 | Ref  1.26 (1.07-1.47) | Ref  0.005 |
| **Occupation**  Unemployed/home duties  Employed  Retired  Student | Ref  1.05 (0.79-1.40)  0.39 (0.08-1.84) 1.69 (0.75-3.78) | Ref  0.73  0.23  0.20 | Ref  1.04 (0.82-1.31)  0.79 (0.17-3.74) 1.50 (0.78-2.85) | Ref  0.76  0.77  0.22 | Ref  1.21 (0.96-1.52)  0.98 (0.21-4.60) 2.00 (1.00-3.98) | Ref  0.11  0.98  0.049 | Ref  0.91 (0.75-1.10)  0.29 (0.09-0.94) 1.10 (0.67-1.83) | Ref  0.34  0.040  0.70 | Ref  0.99 (0.82-1.20)  0.60 (0.19-1.91) 0.88 (0.54-1.45) | Ref  0.90  0.39  0.62 |
| **Monthly income**  < 1450 NIS  ≥ 1450 NIS | Ref  1.30 (1.01-1.67) | Ref  0.038 | Ref  1.11 (0.90-1.38) | Ref  0.33 | Ref  1.16 (0.94-1.43) | Ref  0.16 | Ref  0.88 (0.74-1.05) | Ref  0.17 | Ref  0.92 (0.77-1.09) | Ref  0.34 |
| **Residency**  Gaza Strip  WBJ | Ref  0.75 (0.59-0.96) | Ref  0.021 | Ref  0.58 (0.47-0.72) | Ref  0.001> | Ref  0.64 (0.52-0.78) | Ref  0.001> | Ref  1.00 (0.84-1.19) | Ref  0.97 | Ref  0.78 (0.66-0.93) | Ref  0.005 |
| **Having a chronic disease**  No  Yes | Ref  1.15 (0.90-1.47) | Ref  0.27 | Ref  1.09 (0.89-1.34) | Ref  0.41 | Ref  1.16 (0.94-1.42) | Ref  0.16 | Ref  1.04 (0.88-1.24) | Ref  0.62 | Ref  1.16 (0.98-1.37) | Ref  0.09 |
| **Knowing someone with cancer**  No  Yes | Ref  1.47 (1.22-1.78) | Ref  0.001> | Ref  1.19 (1.02-1.40) | Ref  0.026 | Ref  1.06 (0.91-1.24) | Ref  0.44 | Ref  1.34 (1.17-1.53) | Ref  0.001> | Ref  1.03 (0.90-1.17) | Ref  0.70 |
| **Marital status**  Married  Divorced/Widowed | Ref  0.96 (0.67-1.37) | Ref  0.82 | Ref  0.71 (0.53-0.94) | Ref  0.017 | Ref  0.85 (0.64-1.14) | Ref  0.27 | Ref  1.29 (0.99-1.68) | Ref  0.06 | Ref  0.97 (0.75-1.25) | Ref  0.81 |
| **Site of data collection**  Public spaces  Hospitals  Primary healthcare centers | Ref  1.56 (1.25-1.95)  1.18 (0.93-1.50) | Ref  0.001>  0.17 | Ref  1.74 (1.44-2.10)  1.38 (1.12-1.69) | Ref  0.001>  0.002 | Ref  1.57 (1.30-1.89)  1.29 (1.05-1.58) | Ref  0.001>  0.015 | Ref  1.55 (1.32-1.82)  1.07 (0.90-1.28) | Ref  0.001>  0.43 | Ref  1.18 (1.01-1.38)  1.03 (0.86-1.23) | Ref  0.041  0.77 |

AOR= adjusted odds ratio, CI= confidence interval, WBJ= West Bank and Jerusalem.
* Adjusted for age-group, parity, educational level, occupation, monthly income, marital status, residency, having a chronic disease, knowing someone with cancer, and site of data collection.

| **characteristic**  Supplementary table 5: Bivariable logistic regression analyzing the association between recognizing breast symptoms and participant characteristics. | **A lump or thickening in the breast** | | **Pain in one of the breasts or armpits** | | **Puckering or dimpling of breast skin** | | **Redness of the breast skin** | |
| --- | --- | --- | --- | --- | --- | --- | --- | --- |
|  | **COR (95% CI)** | **p-value** | **COR (95% CI)** | **p-value** | **COR (95% CI)** | **p-value** | **COR (95% CI)** | **p-value** |
| **Age group**  18 to 39  40 or older | Ref  1.16 (0.92-1.46) | Ref  0.22 | Ref  0.70 (0.62-0.79) | Ref  >0.001 | Ref  1.26 (1.12-1.41) | Ref  >0.001 | Ref  1.36 (1.21-1.53) | Ref  >0.001 |
| **Parity***  Nulliparity  Low multiparity  Grand multiparity | Ref  1.09 (0.68-1.74)  1.18 (0.73-1.90) | Ref  0.72  0.50 | Ref  0.89 (0.70-1.15)  0.67 (0.52-0.86) | Ref  0.38  0.002 | Ref  1.08 (0.85-1.38)  1.21 (0.94-1.54) | Ref  0.54  0.13 | Ref  0.96 (0.75-1.23)  1.06 (0.83-1.36) | Ref  0.75  0.64 |
| **Educational level**  Secondary or below  Post-secondary | Ref  1.67 (1.33-2.09) | Ref  0.001> | Ref  1.33 (1.19-1.48) | Ref  0.001> | Ref  1.37 (1.23-1.54) | Ref  0.001> | Ref  1.00 (0.89-1.11) | Ref  0.93 |
| **Occupation**  Unemployed/home duties  Employed  Retired  Student | Ref  1.08 (0.82-1.43)  0.90 (0.12-6.92)  0.80 (0.59-1.10) | Ref  0.58  0.92  0.17 | Ref  0.97 (0.84-1.11)  4.06 (0.90-18.34)  1.35 (1.13-1.61) | Ref  0.65  0.7  0.001 | Ref  1.13 (0.98-1.29)  2.68 (0.74-9.74)  1.13 (0.95-1.34) | Ref  0.10  0.14  0.16 | Ref  0.84 (0.73-0.97)  0.86 (0.29-2.56)  0.79 (0.67-0.94) | Ref  0.015  0.79  0.007 |
| **Monthly income**  < 1450 NIS  ≥ 1450 NIS | Ref  1.27 (1.02-1.57) | Ref  0.29 | Ref  1.04 (0.93-1.17) | Ref  0.45 | Ref  1.00 (0.89-1.11) | Ref  0.96 | Ref  0.70 (0.63-0.78) | Ref  >0.001 |
| **Residency**  Gaza Strip  WBJ | Ref  0.73 (0.59-0.91) | Ref  0.004 | Ref  1.02 (0.92-1.14) | Ref  0.66 | Ref  0.79 (0.71-0.88) | Ref  >0.001 | Ref  0.67 (0.60-0.75) | Ref  >0.001 |
| **Having a chronic disease**  No  Yes | Ref  1.05 (0.80-1.37) | Ref  0.74 | Ref  0.76 (0.67-0.88) | Ref  >0.001 | Ref  1.02 (0.89-1.17) | Ref  0.80 | Ref  1.11 (0.97-1.27) | Ref  0.14 |
| **Knowing someone with cancer**  No  Yes | Ref  1.47 (1.18-1.82) | Ref  0.001 | Ref  0.89 (1.80-1.00) | Ref  0.045 | Ref  1.13 (1.01-1.26) | Ref  0.03 | Ref  1.04 (0.93-1.16) | Ref  0.50 |
| **Marital status**  Single  Married  Divorced/Widowed | Ref  1.36 (1.08-1.72)  1.81 (1.04-3.15) | Ref  0.009  0.035 | Ref  0.79 (0.69-0.90)  0.73 (0.57-0.94) | Ref  0.001>  0.015 | Ref  0.96 (0.85-1.09)  0.95 (0.74-1.23) | Ref  0.55  0.72 | Ref  1.34 (1.18-1.52)  1.04 (0.81-1.34) | Ref  0.001>  0.74 |
| **Site of data collection**  Public spaces  Hospitals  Primary healthcare centers  COR= crude odds ratio, CI= confidence interval, WBJ= West Bank and Jerusalem. Note: all study participants were included in the multivariable analyses except where indicated by * (only married, divorced, or widowed women were included.) | Ref  1.34 (1.04-1.72)  0.95 (0.73-1.24) | Ref  0.023  0.73 | Ref  0.95 (0.84-1.08)  1.28 (1.10-1.48) | Ref  0.48  0.001 | Ref  1.56 (1.38-1.77)  1.77 (1.53-2.05) | Ref  >0.001  >0.001 | Ref  1.50 (1.32-1.70)  1.60 (1.38-1.84) | Ref  >0.001  >0.001 |

| **Characteristic** | **Discharge or bleeding from the nipple** | | **Nipple rash** | | **Change in the position of the nipple** | | **Pulling in of the nipple** | |
| --- | --- | --- | --- | --- | --- | --- | --- | --- |
|  | **COR (95% CI)** | **p-value** | **COR (95% CI)** | **p-value** | **COR (95% CI)** | **p-value** | **COR (95% CI)** | **p-value** |
| **Age group**  18 to 39  40 or older | Ref  1.27 (1.11-1.45) | Ref  >0.001 | Ref  1.15 (1.03-1.30) | Ref  0.018 | Ref  1.39 (1.23-1.56) | Ref  >0.001 | Ref  1.39 (1.23-1.56) | Ref  >0.001 |
| **Parity***  Nulliparity  Low multiparity  Grand multiparity | Ref  1.10 (0.85-1.43)  1.29 (0.99-1.68) | Ref  0.47  0.06 | Ref  0.87 (0.68-1.12)  0.95 (0.74-1.22) | Ref  0.28  0.71 | Ref  1.09 (0.86-1.39)  1.22 (0.95-1.55) | Ref  0.46  0.12 | Ref  0.86 (0.68-1.10)  1.04 (0.82-1.33) | Ref  0.23  0.75 |
| **Educational level**  Secondary or below  Post-secondary | Ref  1.36 (1.20-1.53) | Ref  0.001> | Ref  1.13 (1.01-1.26) | Ref  0.030 | Ref  1.09 (0.98-1.22) | Ref  0.11 | Ref  1.24 (1.12-1.39) | Ref  0.001> |
| **Occupation**  Unemployed/home duties  Employed  Retired  Student | Ref  1.34 (1.14-1.57)  2.31 (0.51-10.42)  1.17 (0.96-1.41) | Ref  >0.001  0.28  0.12 | Ref  0.94 (0.82-1.08)  2.35 (0.65-8.56)  0.96 (0.81-1.14) | Ref  0.39  0.19  0.66 | Ref  1.00 (0.86-1.14)  5.19(1.15-23.44)  1.02 (0.86-1.21) | Ref  0.90  0.032  0.83 | Ref  1.19 (1.04-1.37)  3.40 (0.93-12.37)  1.08 (0.91-1.28) | Ref  0.013  0.06  0.38 |
| **Monthly income**  < 1450 NIS  ≥ 1450 NIS | Ref  1.27 (1.13-1.44) | Ref  >0.001 | Ref  0.81 (0.72-0.90) | Ref  >0.001 | Ref  0.93 (0.83-1.04) | Ref  0.20 | Ref  0.94 (0.84-1.05) | Ref  0.27 |
| **Residency**  Gaza Strip  WBJ | Ref  0.91 (0.81-1.03) | Ref  0.12 | Ref  0.69 (0.62-0.77) | Ref  >0.001 | Ref  0.77 (0.69-0.85) | Ref  >0.001 | Ref  0.72 (0.65-0.80) | Ref  >0.001 |
| **Having a chronic disease**  No  Yes | Ref  1.18 (1.01-1.37) | Ref  0.037 | Ref  1.02 (0.89-1.17) | Ref  0.80 | Ref  1.28 (1.12-1.46) | Ref  >0.001 | Ref  1.16 (1.02-1.33) | Ref  0.029 |
| **Knowing someone with cancer**  No  Yes | Ref  1.03 (0.92-1.17) | Ref  0.60 | Ref  1.14 (1.02-1.27) | Ref  0.020 | Ref  1.04 (0.93-1.16) | Ref  0.48 | Ref  1.09 (0.98-1.21) | Ref  0.13 |
| **Marital status**  Single  Married  Divorced/Widowed | Ref  1.10 (0.96-1.27)  1.12 (0.85-1.49) | Ref  0.17  0.42 | Ref  1.15 (1.01-1.30)  1.00 (0.78-1.29) | Ref  0.038  0.98 | Ref  1.00 (0.88-1.14)  1.10 (0.85-1.41) | Ref  0.95  0.47 | Ref  1.10 (0.97-1.25)  1.09 (0.85-1.40) | Ref  0.13  0.51 |
| **Site of data collection**  Public spaces  Hospitals  Primary healthcare centers | Ref  1.35 (1.17-1.55)  1.50 (1.28-1.76) | Ref  >0.001  >0.001 | Ref  1.31 (1.15-1.49)  1.41 (1.22-1.63) | Ref  >0.001  >0.001 | Ref  1.44 (1.27-1.63)  1.54 (1.34-1.78) | Ref  >0.001  >0.001 | Ref  1.36 (1.20-1.54)  1.73 (1.50-2.00) | Ref  >0.001  >0.001 |

COR= crude odds ratio, CI= confidence interval, WBJ= West Bank and Jerusalem.
Note: all study participants were included in the multivariable analyses except where indicated by * (only married, divorced, or widowed women were included.)

Supplementary table 6: Bivariable logistic regression analyzing the association between recognizing nipple symptoms and participant characteristics.

| **Characteristic**  Supplementary table 7: Bivariable logistic regression analyzing the association between recognizing other symptoms and participant characteristics. | **Lump or thickening under the armpit** | | **Changes in the shape of the breast or nipple** | | **Changes in the size of the breast or nipple** | | **Unexplained weight loss** | | **Extreme generalized fatigue** | |
| --- | --- | --- | --- | --- | --- | --- | --- | --- | --- | --- |
|  | **COR (95% CI)** | **p-value** | **COR (95% CI)** | **p-value** | **COR (95% CI)** | **p-value** | **COR (95% CI)** | **p-value** | **COR (95% CI)** | **p-value** |
| **Age group**  18 to 39  40 or older | Ref  1.58 (1.34-1.88) | Ref  >0.001 | Ref  1.21 (1.05-1.40) | Ref  0.008 | Ref  1.25 (1.09-1.44) | Ref  0.002 | Ref  1.35 (1.20-1.53) | Ref  >0.001 | Ref  0.97 (0.86-1.09) | Ref  0.59 |
| **Parity***  Nulliparity  Low multiparity  Grand multiparity | Ref  1.12 (0.80-1.56)  1.16 (0.83-1.62) | Ref  0.50  0.38 | Ref  0.94 (0.70-1.26)  1.10 (0.82-1.48) | Ref  0.68  0.51 | Ref  0.89 (0.67-1.19)  1.02 (0.76-1.37) | Ref  0.44  0.88 | Ref  1.10 (0.86-1.41)  1.28 (1.00-1.64) | Ref  0.43  0.047 | Ref  1.03 (0.81-1.31)  1.00 (0.79-1.28) | Ref  0.82  0.97 |
| **Educational level**  Secondary or below  Post-secondary | Ref  1.31 (1.13-1.52) | Ref  0.001> | Ref  1.34 (1.17-1.53) | Ref  0.001> | Ref  1.23 (1.08-1.40) | Ref  0.002 | Ref  1.00 (0.89-1.11) | Ref  0.94 | Ref  1.15 (1.03-1.28) | Ref  0.014 |
| **Occupation**  Unemployed/home duties  Employed  Retired  Student | Ref  1.11 (0.91-1.35)  1.04 (0.23-4.70)  0.65 (0.53-0.80) | Ref  0.29  0.96  >0.001 | Ref  1.01 (0.86-1.19)  1.61 (0.36-7.27)  0.90 (0.74-1.10) | Ref  0.90  0.54  0.30 | Ref  1.17 (0.99-1.39)  1.73 (0.38-7.80)  0.85 (0.70-1.03) | Ref  0.06  0.48  0.10 | Ref  0.91 (0.79-1.04)  0.55 (0.18-1.63)  0.80 (0.68-0.95) | Ref  0.17  0.28  0.012 | Ref  1.01 (0.88-1.16)  0.86 (0.29-2.56)  1.17 (0.98-1.39) | Ref  0.92  0.78  0.08 |
| **Monthly income**  < 1450 NIS  ≥ 1450 NIS | Ref  1.17 (1.01-1.35) | Ref  0.038 | Ref  0.95 (0.84-1.09) | Ref  0.47 | Ref  0.92 (0.81-1.05) | Ref  0.24 | Ref  0.93 (0.83-1.04) | Ref  0.22 | Ref  0.82 (0.73-0.92) | Ref  >0.001 |
| **Residency**  Gaza Strip  WBJ | Ref  0.85 (0.74-0.99) | Ref  0.032 | Ref  0.66 (0.58-0.75) | Ref  >0.001 | Ref  0.71 (0.62-0.81) | Ref  >0.001 | Ref  0.96 (0.86-1.07) | Ref  0.42 | Ref  0.79 (0.71-0.88) | Ref  >0.001 |
| **Having a chronic disease**  No  Yes | Ref  1.36 (1.12-1.66) | Ref  0.002 | Ref  1.06 (0.90-1.25) | Ref  0.46 | Ref  1.12 (0.96-1.32) | Ref  0.16 | Ref  1.28 (1.11-1.48) | Ref  0.001 | Ref  1.07 (0.94-1.23) | Ref  0.32 |
| **Knowing someone with cancer**  No  Yes | Ref  1.48 (1.28-1.72) | Ref  >0.001 | Ref  1.15 (1.01-1.31) | Ref  0.034 | Ref  1.05 (0.93-1.20) | Ref  0.42 | Ref  1.32 (1.18-1.47) | Ref  >0.001 | Ref  1.04 (0.93-1.16) | Ref  0.50 |
| **Marital status**  Single  Married  Divorced/Widowed | Ref  1.70 (1.45-2.00)  1.71 (1.21-2.42) | Ref  0.001>  0.003 | Ref  1.23 (1.06-1.43)  0.86 (0.65-1.14) | Ref  0.006  0.29 | Ref  1.25 (1.08-1.44)  1.08 (0.81-1.44) | Ref  0.003  0.61 | Ref  1.22 (1.08-1.39)  1.64 (1.25-2.13) | Ref  0.002  0.001> | Ref  0.92 (0.81-1.05)  0.89 (0.69-1.14) | Ref  0.21  0.35 |
| **Site of data collection**  Public spaces  Hospitals  Primary healthcare centers | Ref  1.56 (1.32-1.85)  1.32 (1.10-1.60) | Ref  >0.001  0.003 | Ref  1.53 (1.32-1.77)  1.42 (1.20-1.68) | Ref  >0.001  >0.001 | Ref  1.45 (1.25-1.68)  1.31 (1.11-1.55) | Ref  >0.001  0.001 | Ref  1.61 (1.42-1.83)  1.05 (0.91-1.21) | Ref  >0.001  0.48 | Ref  1.08 (0.96-1.23)  0.99 (0.85-1.14) | Ref  0.21  0.85 |

COR= crude odds ratio, CI= confidence interval, WBJ= West Bank and Jerusalem.
Note: all study participants were included in the multivariable analyses except where indicated by * (only married, divorced, or widowed women were included.)
