# Supplementary material for: Women’s awareness of breast cancer symptoms: a national cross-sectional study from Palestine
Source: BMC Public Health. 2022 Apr 21;22:801. doi: 10.1186/s12889-022-13224-7 (PMC9027340; doi:10.1186/s12889-022-13224-7)
Supplement: Supplementary file 2 — Additional file 2. [file 12889_2022_13224_MOESM2_ESM.docx]

**Breast Cancer Awareness Questionnaire**

Serial number: ………. Location: …………. Governorate: ……….

- Do you know anyone who has cancer? 🞏 No 🞏 Yes

If yes, what type of cancer they have?

🞏 breast and/or ovarian cancer 🞏 Other cancer 🞏 Both

**Awareness of Breast Cancer Symptoms**

| The following may or may not be warning signs for breast cancer. We are interested in your opinion: | | | | | | |
| --- | --- | --- | --- | --- | --- | --- |
|  | **1= Strongly Disagree** | **2= Disagree** | **3= Not Sure** | **4= Agree** | **5= Strongly agree** |  |
| 1. A change in the position of your nipple |  |  |  |  |  |  |
| 1. Pulling in of your nipple |  |  |  |  |  |  |
| 1. Pain in one of your breasts or armpit |  |  |  |  |  |  |
| 1. Puckering or dimpling of your breast skin |  |  |  |  |  |  |
| 1. Discharge or bleeding from your nipple |  |  |  |  |  |  |
| 1. A Lump or thickening in your breast |  |  |  |  |  |  |
| 1. Nipple rash |  |  |  |  |  |  |
| 1. Redness of your breast skin |  |  |  |  |  |  |
| 1. Lump or thickening under your armpit |  |  |  |  |  |  |
| 1. Changes in the size of your breast or nipple |  |  |  |  |  |  |
| 1. Changes in the shape of your breast or nipple |  |  |  |  |  |  |
| 1. Extreme fatigue |  |  |  |  |  |  |
| 1. Unexplained weight loss |  |  |  |  |  |  |

**Sociodemographic Data**

- Age: …….. years
- Age for first menstrual cycle (menarche): ………. years
- Marital status: 🞏 Single 🞏 Married 🞏 Divorced 🞏 Widowed

**If married, divorced or widowed:**

- Parity: …………
- Highest level of education: 🞏 Illiterate 🞏 Primary 🞏 Prep 🞏 Secondary

🞏 Bachelor degree 🞏 Postgraduate studies

- Occupation: 🞏 Housewife/unemployed 🞏 Employed 🞏 Retired 🞏 Student
- Monthly income (NIS): ………………
- Place of residency: 🞏 West Bank and Jerusalem 🞏 Gaza Strip
- Do you have any chronic disease? 🞏 No 🞏 Yes
